# Intercropping with Shrub Species That Display a ‘Steady-State’ Flowering Phenology as a Strategy for Biodiversity Conservation in Tropical Agroecosystems

**DOI:** 10.1371/journal.pone.0090510

**Published:** 2014-03-05

**Authors:** Valerie E. Peters

**Affiliations:** Odum School of Ecology, University of Georgia, Athens, Georgia, United States of America; Estacion Experimental de Zonas Áridas (CSIC), Spain

## Abstract

Animal species in the Neotropics have evolved under a lower spatiotemporal patchiness of food resources compared to the other tropical regions. Although plant species with a steady-state flowering/fruiting phenology are rare, they provide predictable food resources and therefore may play a pivotal role in animal community structure and diversity. I experimentally planted a supplemental patch of a shrub species with a steady-state flowering/fruiting phenology, *Hamelia patens* Jacq., into coffee agroforests to evaluate the contribution of this unique phenology to the structure and diversity of the flower-visiting community. After accounting for the higher abundance of captured animals in the coffee agroforests with the supplemental floral resources, species richness was 21% higher overall in the flower-visiting community in these agroforests compared to control agroforests. Coffee agroforests with the steady-state supplemental floral patch also had 31% more butterfly species, 29% more hummingbird species, 65% more wasps and 85% more bees than control coffee agroforests. The experimental treatment, together with elevation, explained 57% of the variation in community structure of the flower-visiting community. The identification of plant species that can support a high number of animal species, including important ecosystem service providers, is becoming increasingly important for restoration and conservation applications. Throughout the Neotropics plant species with a steady-state flowering/fruiting phenology can be found in all aseasonal forests and thus could be widely tested and suitable species used throughout the tropics to manage for biodiversity and potentially ecosystem services involving beneficial arthropods.

## Introduction

The relatively low degree of spatiotemporal variation in flower and fruit availability in Neotropical forests has favored the evolution of more diverse communities of frugivores and pollinators compared to the other tropical regions [1]–[4]. One unique component of the Neotropical flora that contributes to reducing the spatiotemporal patchiness of resources is an Andean-centered radiation of epiphytes, understory shrubs, and palmetto-like monocots [5]. Not only does this group contribute to providing a more abundant and species-rich food resource base in the Neotropics, but also some plant species in this group provide their pollinators or dispersal agents with a year-round food supply. This is accomplished either by a single species through a continual [6] or ‘steady-state’ [7] flowering/fruiting phenology (hereafter, both terms are used interchangeably, as different authors have presented these terms to describe the same phenology) at the individual or population level [8] or at the guild level with individual species in the guild having a staggered phenology [1]. The overall effect of either strategy is to maintain their animal dispersers and pollinators in residence in the community [9].

The importance of plant species with extended, continuous or steady-state resource production has been most broadly studied for their role in maintaining frugivore populations during times of resource scarcity [10]–[14] and to a lesser extent for their role (a) in the evolution of a more diverse specialized pollinator group in the Neotropics [4] and (b) in dampening fluctuations in pollinator abundances [15]. The concept of ‘bridging plants’, which does not include the duration of flowering, has been employed to describe plant species that could potentially be used to restore pollinator communities successfully because they provide nectar and pollen resources during otherwise resource-limited times [16], [17]. Only recently, though, have studies begun to identify and experimentally test bridging species [17].

Plant species with continuous resource production at the individual level are rare and most frequently early successional species [18], [19]. In addition, some Neotropical plant species either alter resource production or only produce resources in early successional habitats, such as treefall gaps, where resource density is higher than in undisturbed forest [1], [11]. Not only do these habitats play a key role in structuring Neotropical communities [20], [21] but they also provide critical resources to animal species, especially during times of resource scarcity [11], [22]. However, early successional habitat may not be sufficient in landscapes where development and agriculture are the dominant land use, and where natural disturbance regimes are suppressed [23]. Restoring early successional habitat or characteristics of early successional habitats in landscapes where they are lacking due to human impact may contribute to the conservation of biodiversity [24].

The role that the early successional habitat characteristic of steady-state resource production has in supporting Neotropical organisms has not been evaluated. While ecologists working in undisturbed Neotropical forests still struggle to accurately estimate how animal populations naturally fluctuate in response to natural variation in food resources [25], agroecologists must move ahead to experimentally test potential management practices that can decrease the spatiotemporal patchiness of food resources in fragmented landscapes. The community-wide demand for any given resource should be frequency-dependent and inversely related to the number of alternative resources that are simultaneously available [12]. Therefore, the predictable and extended food resources provided by any plant species with a steady-state phenology, regardless of resource quality, should have a community-wide impact, especially in agricultural lands where gaps in food availability are more frequent and of longer duration. Despite this, plant species with a steady-state phenology at the individual level have not been studied for their contribution to biodiversity conservation and structuring animal communities in agricultural lands. To determine whether steady-state resource production is important for species in fragmented landscapes, I conducted a manipulative field study in coffee agroforests. The coffee agroforest provides an ideal framework for experimental manipulation [26] and can be managed for biodiversity conservation [27]–[30]. I hypothesized that coffee agroforests with intercropped steady-state flowering shrubs would host a greater diversity and abundance of flower-visiting species compared to control coffee agroforests. Specifically, I selected one shrub species, *Hamelia patens* Jacq., to exemplify the steady-state flowering phenology, experimentally planted a supplemental patch of this species in several coffee agroforests, and quantified the response of the flower-visiting community, i.e. hummingbirds, butterflies, bees and wasps.

## Materials and Methods

### Ethics statement

All research in this study conforms with the legal requirements for field work in Costa Rica and the United States of America. A permit to conduct research in Costa Rica was approved by the Costa Rican Ministerio de Ambiente y Energia (MINAE; Permit Number 014-2010-ACAT). Hummingbird mist-net capture and handling techniques were reviewed and approved by the University of Georgia’s Animal Care and Use Committee (A2008 03-061-Y3-A0). All land accessed for this study was privately owned and all six landowners gave permission for this study to be conducted on their land. No listed endangered or protected species were involved in this study.

### Study sites

Study sites were located in the Monteverde region of the Puntarenas province of Costa Rica (10°N, 84°W; Fig. S1). Six coffee agroforests that shared similar management regimes and were in close proximity to the University of Georgia’s research station in the Upper San Luis Valley (elevation 925–1100 m) were selected. The six agroforests were intercropped with *Musa*, *Citrus*, and *Psidium* spp., had high shade tree diversity (19–23 tree species ha^−1^) and a high shade canopy cover (62–80% canopy cover). The herbaceous ground cover was removed via machete, monthly, during all months of the rainy season (May to November), and farmers did not use pesticides. Each agroforest was separated by at least 100 m from another agroforest. Extensive mark-recapture data from the agroforests revealed that most bird species, including hummingbirds, did not move among the agroforests [31], [32].

### Study species

*Hamelia patens* Jacq. (Rubiaceae) occurs in secondary growth from Mexico to Bolivia. Demonstrating tolerance of a wide range of environmental conditions, *H. patens* has been recorded from 0 to 2000 m elevation in Costa Rica and its phenology has been documented from lowland wet, lowland dry and cloud forests in Costa Rica [18], [33]. In lowland wet forests of Costa Rica where only about 7% of shrub and treelet species exhibit continuous flowering, *H. patens* individuals in secondary growth had flowers throughout the year [18], [34]. In these forests each inflorescence produces a total of 0 to 5 open flowers per day at an average rate of 1.5 per day, producing from 30 to more than 100 flowers over its "lifetime” [35]. In Costa Rican cloud forests, 33% of shrub and treelet species were identified as extended flowerers, although *H. patens* was the only species recorded with flowers during all months of the year. In contrast, *H. patens* plants in Costa Rican lowland dry forests flower only during early wet season months [18]. In the landscape where our study was conducted, *H. patens* was the only plant species with flowers and fruit during all months of the year.

**Figure 1:**
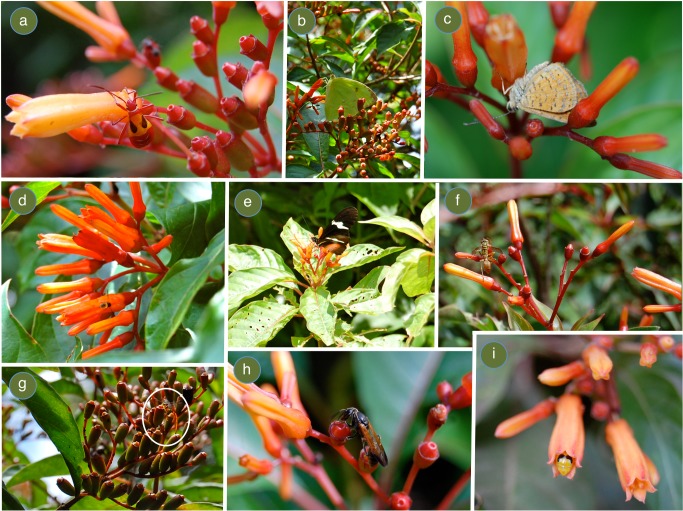
*Hamelia patens* attracts a generalist assemblage of insect species in the study area. (a) Nectar-robbing assassin bug and ant species, (b) *Aphrissa* sp. foraging for nectar, (c) *Calephelis* sp. robbing nectar, (d) Diptera species robbing nectar, (e) *Heliconius* sp. foraging for nectar, (f-h) Wasp species were observed to systematically visit *H. patens* floral ovaries after flowers had fallen off, (i) Coleoptera species inside corolla.

**Figure 2:**
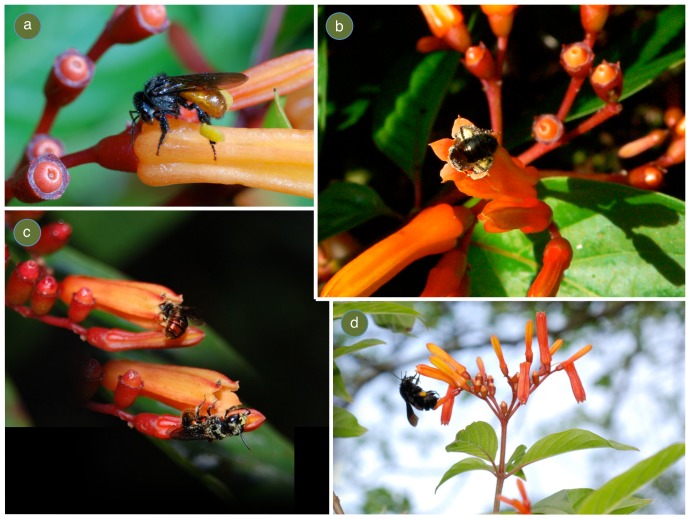
Bees pollinating *Hamelia patens*. (a) *Trigona fulviventris* with collected *H. patens* pollen (b) Halictidae species emerging from *H. patens* corolla, covered in pollen (c) *Ceratina* species, emerging from corolla and covered in pollen (d) *Bombus pullatus* with collected *H. patens* pollen.

Hypothesized selective forces for plant species with the continuous flowering and fruiting strategy are (1) its primary pollen vector, hummingbirds, and (2) its association with early successional habitats, which are ephemeral, because the likelihood of successful colonization will increase with more frequent seed production [19]. In the literature, *H. patens* has been most notably associated with hummingbird pollination [35], [36], and its flowers which are odorless, orange, narrow and tubular fit the bird-pollination syndrome. However, pollination syndromes can lead researchers to focus only on floral visitors that conform to the ‘correct’ pollinator. In reality, though, flowers conforming to a particular syndrome can receive visits from opportunistic insects belonging to different orders that contribute to the fitness of the plant [37]. In fact, *H. patens* individuals across the fragmented landscape where this study was conducted attracted a generalist assemblage of pollinators and floral visitors (Fig. 1), with some moving visibly more pollen than the hummingbird visitors (Fig. 2). However, the aim of this study was not to evaluate pollinator effectiveness, but instead to evaluate the impact of plant species with a continuous flowering strategy on the animal community that uses floral resources in agroecosystems.

### Experimental design

In May 2007 fifteen *H. patens* shrubs were experimentally planted within a 400-m^2^ plot in three out of the six coffee agroforests selected for the study. All shrubs were planted via either (1) transplanting shrubs < 1m from nearby roadsides where they were growing wild or (2) flagging seedlings naturally occurring within the coffee farm so that the farmer would not remove them when weeding in between coffee rows. Agroforests with supplementation plots were not selected randomly but instead determined by the farmer’s willingness to permit me to supplement their farms with *H. patens* patches. Moran’s *I* analyses were used to test for spatial dependence in the response variables among sample locations. No spatial autocorrelation in the response variables after treatments was found (Table 1). Prior observation of *H. patens* plants in the region revealed that individuals located in shaded conditions produced few flowers, and therefore *H. patens* plots were located both close to the middle of the coffee agroforests and in full sun to maximize the number of flowers. Individual *H. patens* plants began producing flowers by August 2007. Floral resource availability (i.e. the number of all open, non-coffee flowers, including *H. patens* flowers) was estimated for all trees, shrubs and herbaceous plants within each 1–2 ha agroforest (Table S1) for each month, except for September, from February to December 2008.

**Table 1:** Moran’s I statistics for testing spatial autocorrelation in response variables.

Variable	Observed value	Expected value	Standard deviation	p-value
Wasp richness	–0.016	–0.034	0.032	0.57
Wasp abundance	–0.001	–0.034	0.033	0.31
Butterfly richness	–0.015	–0.043	0.041	0.48
Butterfly abundance	–0.041	–0.043	0.041	0.95
Hummingbird richness	–0.021	–0.008	0.008	0.10
Hummingbird abundance	–0.016	–0.008	0.008	0.30
Bee richness	–0.004	–0.029	0.028	0.37
Bee abundance	–0.004	–0.029	0.027	0.37

### Bird and insect sampling

Hummingbirds were the only bird pollinators in the study area that used floral resources of *H. patens*. Mist-nets were used to quantify hummingbird abundance and species richness in the coffee agroforests. Hummingbirds were captured during three sampling periods from 23 March to 22 May 2009, 14 July to 6 August 2009 and 9 June to 27 July 2010 using 30-mm, 34-mm and 60-mm mesh mist-nets. All coffee agroforests were sampled in each of the three sampling periods. Three mist-nets were placed along windbreaks in each agroforest for 5 to 11 days per sampling period. Nets were opened daily from 700 to 1400 hours except during periods of heavy rain. Hummingbird species richness and abundances were totaled on a daily basis for each agroforest.

Insects were sampled with Malaise traps, with one trap placed near the center of each coffee agroforest per sampling period. Malaise traps were left open for 15 days and sampling was conducted in July 2008, Nov 2008, May 2009, July 2009, Nov 2009, and July 2010. Each agroforest was sampled during each of the six sampling periods. At the end of each sampling period, all insects from the Malaise traps were collected and identified to order in the laboratory. All captured butterflies were identified to species and categorized as either nectar-feeding or fruit-feeding, based on DeVries [38], [39]. Among Hymenoptera the wasps were distinguished from all other families, and were classified into morphospecies. Identification to morphospecies-level has been shown to serve as a good proxy in the estimation of species richness [40]. Although identifications to lower taxonomic categories would have been desirable, the large number of insects precluded greater taxonomic precision. Captured bees were identified to species or genus. Because bees are the most effective pollinators of coffee [27], they were analyzed in a separate paper that focused on assessing the potential role of sowing plants with a steady-state flowering phenology into agricultural lands to increase coffee yield [41]. The potential benefit of adding plant species with a steady-state flowering phenology into agricultural lands for (a) biodiversity conservation and (b) the ecosystem service of pollination were evaluated separately, as both are important goals of management in agricultural lands, but management actions will not always synergistically improve both biodiversity and ecosystem services [31], [42]. However, because this paper deals with conservation of the flower-visiting community as a whole, some new analyses of bees not included in the previous publication are presented, such as (a) habitat specificity, (b) species evenness and (c) species richness after removing the effect of abundance (see Statistical analyses section below for details of these analyses), and bees are also included in all analyses where the variable is ‘flower-visiting community’.

### Statistical analyses

Generalized linear and linear mixed models were used to compare the number of open, non-coffee flowers, open *H. patens* flowers, and hummingbird abundance and species richness between treatments, hereafter H+ for agroforests with supplemental plantings and C for control agroforests. For hummingbirds, models were fit by the Laplace approximation with a Poisson distribution in R version 2.11.1, package ‘lme4’ [43]. All models included treatment as a fixed effect and site and sampling period within site as random effects. To test for statistical significance of the treatment effect, a likelihood ratio test was performed to compare models with and without the treatment as an explanatory variable. To account for overdispersion in the model for hummingbird abundance it was necessary to add an observation-level random effect.

Generalized linear (GLM) and linear models (LM) were used to test how insect groups responded to the experimental plantings of *H. patens*. Models included site as the explicit error term and sampling period as the within error term. GLM and LM was used for malaise trap data because (a) the data were balanced and (b) the sampling phase (within) error term was larger than the site (source) error term, which causes mixed models to set the site error term to zero thus providing an unreliable estimate of the treatment effect. GLMs were fit with a Poisson distribution, and when LMs were used, data were log transformed to meet the conditions of normality.

Likelihood ratio tests comparing models with and without the linear variables of the X-Y coordinates of the plots were used to assess whether there was any linear dependency in the placement of the experimentally applied treatments. Likelihood ratio test results indicate that adding linear variables to the models did not improve model fit (Table S2). Results were not affected by the inclusion of the X-Y coordinates in the models either (Table S3).

Sample-based rarefaction curves were generated with EstimateS version 8.2.0 [44] to compare butterfly, wasp and hummingbird species richness between the treatments. Curves were calculated from 100 randomizations of sample order, without sample replacement. For butterflies and wasps, abundance-based data from Malaise trap capture were used to estimate species richness and a sample represents one 15-day period. For hummingbirds, incidence-based data from mist-net capture were used to estimate species richness and a sample represents one day. The sample-based rarefaction curves for butterflies and wasps were rescaled to the number of individuals to compare species richness between treatments [45], whereas hummingbird rarefaction curves, scaled by sample, compare species density between treatments. Rarefaction curves are presented with the Mao Tau estimate and 95% confidence intervals so that statistical comparisons can be made between treatments.

To remove the effect of abundance on the estimates of species richness in C and H+ agroforests, sample-based randomizations were used. This procedure shuffled all samples across control and treatment agroforests, retaining the number of observed samples during each randomization, and tests whether or not the observed species richness in C and H+ agroforests could have been obtained by random allocation of samples among treatments. Pseudo F-statistics were calculated from the randomized species richness values and ranked F-statistics were compared to the observed to determine P-values [46]. The same procedure was also carried out to compare the relative abundance of species, or evenness, between C and H+ agroforests. Species evenness was calculated by dividing the Shannon diversity index for each site by the natural logarithm of the site’s species richness.

A distance-based RDA (Redundancy Analysis), using the Bray-Curtis distance measure with the capscale function in the R version 2.11.1, package ‘vegan’, was performed to examine variation in community structure of the entire flower-visiting assemblage, including hummingbirds, butterflies, wasps and bees. The analysis was focused on evaluating whether the relationship between the experimental plantings and community structure was greater than what would be expected by chance, and elevation was included in the model because although the experiment was designed to minimize the effect of confounding environmental variables other than the treatment, a slight elevation gradient (925–1100 m elevation) existed among the sites.

Finally, habitat specificity was estimated to determine the number of species out of the total species pool that was more frequently associated with either the H+ or the C agroforests. Habitat specificity was calculated using an area unweighted index calculation that divides the number of species in each treatment by the harmonic mean of species abundances, or by the harmonic mean of the total number of samples for which each species is present [47]. Samples were then randomized to test whether the observed specificity among H+ and C sites could have also been expected by a random allocation of samples among treatments. The randomization procedure shuffled all samples across treatments with the constraint that the number of samples randomly allocated to each treatment was the same as the observed number of samples. Observed values of habitat specificity were then compared to the null distribution to determine whether observed values were significantly different than those expected by chance. Unlike the pseudo F-statistic, this test is two-tailed because observed specificity can either be higher or lower than the expected [48].

## Results

### Flowering phenology

**Figure 3:**
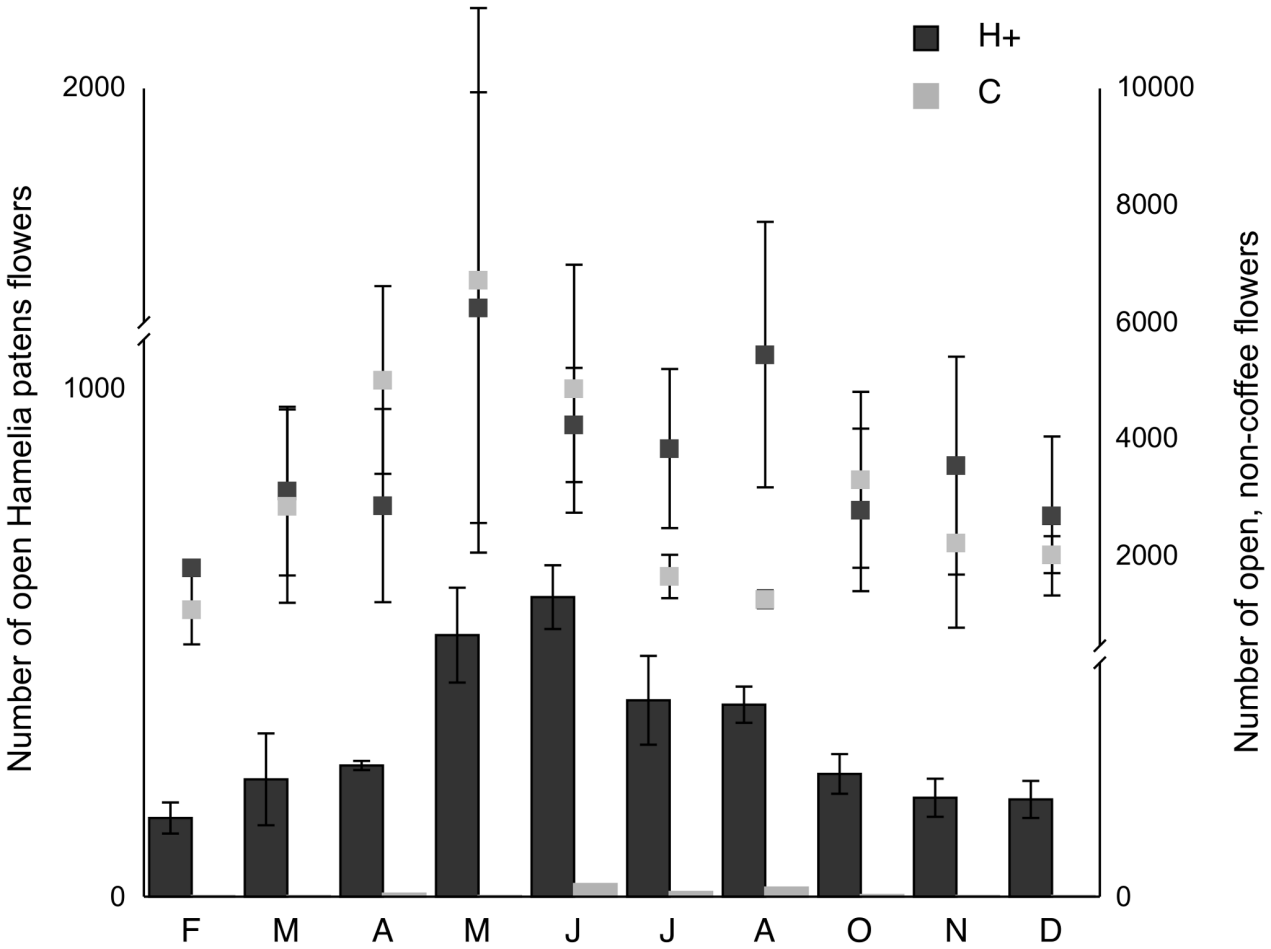
Seasonality of floral resources in coffee agroforests. *Hamelia patens* blooms (first y-axis) and open, non-coffee flowers (second y-axis). H+ represents agroforests with supplemental *H. patens* plantings and C represents control agroforests.

The coefficient of variation (CV) in monthly floral resource availability showed that patchiness in floral resource availability was higher in coffee agroforests without the supplemental patch of *H. patens* (0.88, 0.96 and 1.05) compared to the agroforests with the experimental plantings (0.56, 0.57 and 0.65). All coffee agroforests had >1000 non-coffee flowers available monthly for at least 80% of months for which floral resource availability was estimated. Treatment and control agroforests did not differ in the mean number of open, non-coffee flowers (Likelihood ratio  = 0.34, *P* = 0.56) but did differ in the number of open *H. patens* flowers (Likelihood ratio  = 13.18, *P* = 0.0002); (Fig. 3).

### Flower-visiting community

**Figure 4:**
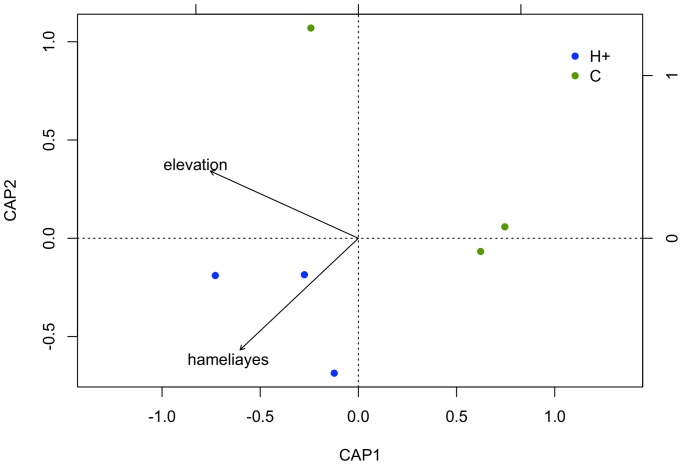
Ordination of the flower-visiting community in coffee agroforests. Distance-based Redundancy Analysis (RDA) of the entire flower-visiting community in coffee agroforests in Costa Rica. H+ represents agroforests with supplemental *H. patens* plantings and C represents control agroforests. The ordination model included elevation and the presence of *H. patens* (represented by hameliayes arrow in graph) supplemental patches (*P* = 0.015).

A total of 7174 potential flower visitors representing 278 species, including hummingbirds, butterflies, wasps and bees, were captured across all H+ and C agroforests during this study. Almost twice as many individuals were captured in H+ agroforests compared to C agroforests, and after accounting for the difference in abundance, species richness of the flower-visiting community was significantly higher in H+ compared to C agroforests (Table 2). The presence of the *H. patens* supplemental floral resource patch was also found to be a significant factor influencing the composition of the flower-visiting community, with elevation and treatment together explaining 57% of the variation in structure of the flower-visiting community (*P* = 0.015, Fig. 4).

**Table 2:** Total abundance, mean total species richness, species evenness and habitat specificity measures for flower-visiting community in coffee agroforests in Costa Rica.

Response variable	H+^a^	C^b^	F	P
**Community** c				
Abundance	4354	2820		
Species richness	150	124	9.66	0.02
Species evenness	0.78	0.78	0.01	0.93
Habitat specificity	157.7	120.3		*
**Hummingbirds**				
Abundance	89	23		
Species richness	5	3	4	0.06
Species evenness	0.93	0.59	1.30	0.22
Habitat specificity	4.7	2.3		*
**Butterflies**				
Abundance	412	530		
Species richness	24	18.3	6.28	0.10
Species evenness	0.73	0.60	12.37	0.10
Habitat specificity	29.9	19.1		*
**Wasps**				
Abundance	3030	1832		
Species richness	87.3	74	3.23	<0.01
Species evenness	0.74	0.76	0.24	0.54
Habitat specificity	86.6	72.4		
**Bees**				
Abundance	823	435		
Species richness	34	28.6	6.92	0.17
Species evenness	0.78	0.79	0.24	0.43
Habitat specificity	36.5	26.5		

a Coffee agroforests with supplemental *H. patens* patch.

b Coffee agroforests without *H. patens.*

c Flower- visiting community includes hummingbirds, butterflies, wasps, and bees.

*Denotes that the observed habitat specificity was significantly different than the expected for both H+ and C sites.

**Figure 5:**
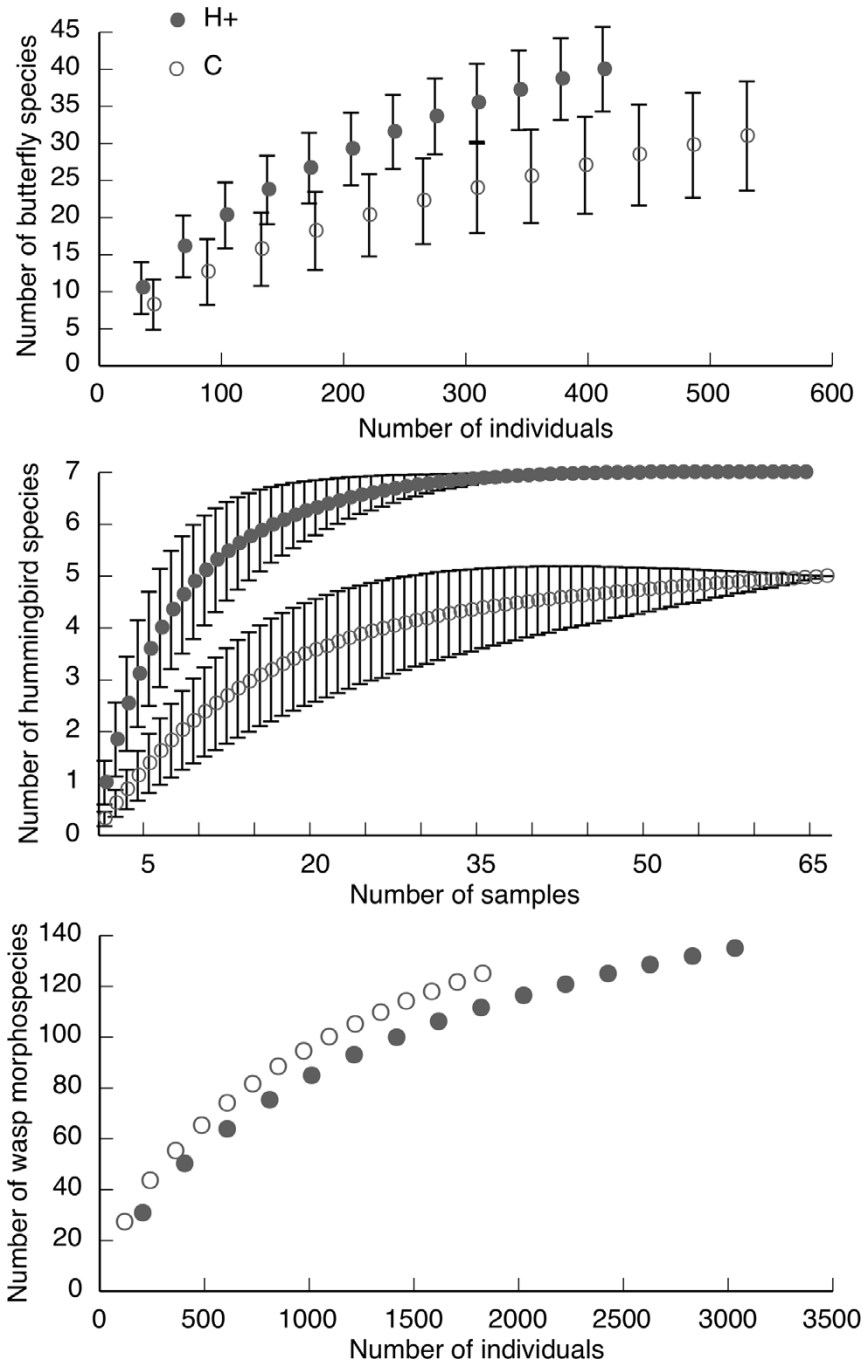
Sample-based rarefaction curves of control and treatment coffee agroforests. Curves compare treatment (H+; supplemental steady-state floral resources) and control (C) coffee agroforests using Mao Tau expected richness in EstimateS. Rarefaction was performed on presence/absence data for hummingbirds (a) and abundance data for butterflies (b) and wasps (c). Curves were rescaled by the number of individuals for butterflies and wasps to compare species richness between agroforest types, and show the mean ± 95% CI. Non-overlapping CI show statistically significant group differences.

A total of 112 hummingbirds from seven species were captured across both types of coffee agroforests. Both hummingbird abundance and species richness were greater in H+ coffee agroforests compared to C coffee agroforests (Table 3). After accounting for the effect of abundance on richness, the slightly higher hummingbird species richness in H+ agroforests was not statistically significant (*P* = 0.06, Table 2). However, species richness curves scaled by sample period showed higher species richness in H+ coffee agroforests (7 species) compared to C coffee (5 species) agroforests (Fig. 5). The number of open, non-coffee flowers of all species in an agroforest was not a significant variable in the models for either hummingbird richness or abundance (richness: likelihood ratio test  = 3.0, *P* = 0.08; abundance: likelihood ratio test  = 2.28, *P* = 0.13).

**Table 3:** Flower-visiting community in coffee agroforests in Costa Rica, comparing agroforests with and without experimentally planted shrubs with a steady-state flowering phenology.

	Mean ± SE		Model	
Response variable	H+^a^	C^b^		*P*
**Hummingbirds**				
Species richness	1.02±0.13	0.32±0.08	Likelihood ratio	0.015
Abundance	1.39±0.26	0.35±0.09	Likelihood ratio	0.006
**Butterflies**				
Overall species richness	10.67±0.93	8.33±1.02	GLM	0.04
Nectarivore species richness	7.75±0.83	5.42±0.87	GLM	0.01
Frugivore species richness	2.92±0.34	2.92±0.34	GLM	0.97
Overall abundance	34.1±4.4	44.2±8.5	LM	0.57
Nectarivore abundance	15.8±2.4	16.8±5.1	LM	0.41
Frugivore abundance	18.2±3.5	27.3±4.6	LM	0.15
**Wasps**				
Morphospecies richness	30.9±3.6	27.3±2.8	LM	0.055
Abundance	202±46.0	122.1±26.4	LM	0.045

a Coffee agroforests with supplemental *H. patens* patch.

b Coffee agroforests without *H. patens.*

A total of 942 butterflies from 49 species were captured across both types of coffee agroforests. Overall butterfly richness and richness within the nectar-feeding butterfly guild were both higher in H+ agroforests, but there was no difference between agroforest types for the fruit-feeding butterfly guild (Table 3). Overall butterfly abundance and within both feeding guilds was higher, though not significantly so, in C agroforests, however when we removed the most common nectar-feeding species, the generalist *Anthanassa ardys*, from our data the direction of higher abundance shifted to H+ coffee agroforests. After accounting for the effect of abundance on richness, H+ agroforests were not found to have higher butterfly richness compared to C agroforests (Table 2). However, rarefaction curves with 95% CI showed a significantly higher number of butterfly species overall in H+ coffee agroforests compared to C agroforests (Fig. 5). Butterfly richness and abundance were not related to the number of open, non-coffee flowers of all plant species in an agroforest (overall butterfly richness: *z* = –1.0, *P* = 0.32; nectarivorous butterfly richness: *z* = –1.3, *P* = 0.21; frugivorous butterfly richness: *z* = 0.1, *P* = 0.90; butterfly abundance: *t* = –0.6, *P* = 0.57).

A total of 4862 wasps from 160 morphospecies were captured in Malaise traps in both types of coffee agroforests. Species richness of wasps was higher in H+ coffee agroforests, however this result was marginally statistically significant (*P* = 0.055, Table 3). Sixty-five percent more wasps were captured in H+ coffee agroforests compared to C coffee agroforests (Table 3). After accounting for the effect of abundance on richness, species richness of wasps was statistically significantly higher in H+ agroforests compared to C agroforests (Table 2). Wasp morphospecies richness and abundance were not related to the number of open, non-coffee flowers of all plant species in an agroforest (richness: *t* = 1.3, *P* = 0.22; abundance: *t* = 0.3, *P* = 0.77).

Bee summary data and results of treatment effects on bees and coffee fruit set were presented in [41]. Pseudo F-tests conducted in this paper, however, remove the confounding effect of abundance on species richness, and show no statistically significant difference in species richness of bees between H+ and C coffee agroforests (Table 2).

### Species evenness and habitat specificity

Species evenness was similar for all taxonomic groups between H+ and C coffee agroforests (Table 2), indicating that the observed increases in abundance in H+ agroforests were evenly distributed across species and not just the result of a few dominant species.

**Figure 6:**
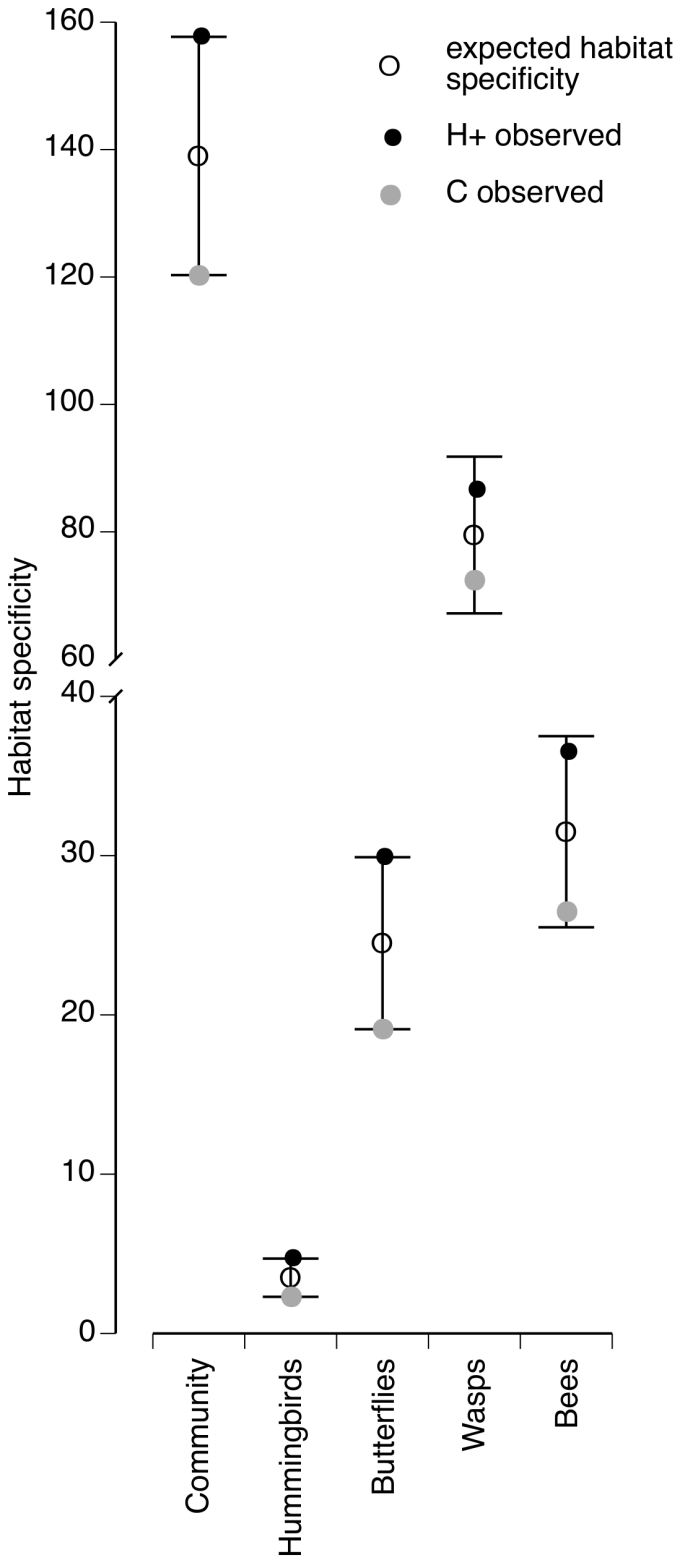
Habitat specificity of flower-visiting species in control and treatment coffee agroforests. Expected habitat specificity and 95% CI were obtained from 1000 sample-based randomizations. Observed habitat specificity is an area unweighted index obtained by dividing the number of species in each treatment by the harmonic mean of species abundances. Observed habitat specificity is shown as either significantly higher or lower than expected by chance if the observed value falls on the outer limits of the 95% CI of the null distribution.

Observed habitat specificity was higher in H+ coffee agroforests for the flower-visiting community and for all groups within the flower-visiting community (Table 2), indicating that agroforests with the supplemental patch of steady-state floral resources represented a larger fraction of the total sampled richness from both types of coffee agroforests. This contribution of higher species richness to the regional species pool was significantly different than what would be expected by chance for the entire flower-visiting community when analyzed as a whole, and for both hummingbirds and butterflies, but not for bees or wasps (Fig. 6).

## Discussion

This study reveals that even when agroecosystems have high plant diversity (approx. 20 tree species ha^−1^), intercropping with plant species with a steady-state flowering phenology can have an impact on the diversity and structure of the flower-visiting community. All coffee agroforests evaluated during this study had a monthly average of >1600 open, non-coffee flowers, and each had >1000 non-coffee flowers available monthly for at least 80% of months for which floral resource availability was estimated. Despite this, the coffee agroforests with the supplemental patch of steady-state floral resources supported 21% more species overall, and this difference was even greater when examining species affinities for H+ versus C coffee agroforests (32% more species showed an affinity for H+ agroforests). Furthermore, the analysis of community composition highlighted the important role that the experimental treatment had in the structure of the flower-visiting community. For example, only 38% of all butterflies captured in C agroforests were species that feed primarily on nectar whereas almost half (46%) of all butterflies captured in H+ coffee agroforests were nectarivorous. In contrast to the majority of plant species, species with a steady-state flowering strategy produce new flowers every day of the year- consequently offering predictable food resources, in a predictable location, and with a considerable amount of nectar becoming available on a daily basis [e.g. one *H. patens* flower can produce up to 50 µl in 24 h [49]; and individual *H. patens* plants in the supplemental patches in this study yielded 20−100 open flowers per day].

Although most previous work with *H. patens* has focused on its relationship with hummingbirds, diffuse interactions in pollination may be far more prevalent than previously recognized [50]. Two other studies have highlighted a more generalist assemblage of pollinators at *H. patens* [51], [52] while others only mention that arthropod visitation does occur [36]. During this study many arthropod species were observed to forage on nectar and pollen resources from *H. patens* flowers, both legitimately and as robbers, as well as to systematically visit other floral related parts, post-flowering (see Fig. 1f-h). Although I was unable to observe which, if any, resources were obtained during these post-flowering visits, I observed ants, flies, and wasps participating in this behavior. It is likely that these resources also display a steady-state phenology if they are related to floral production. One possibility is that just after flowers fall off, nectar is still available at the style base where the nectaries are located [52] and arthropods with short-tongues can take advantage of these resources. One other potential explanation is that the wasp species are searching for *Proctolaelaps kirmsei*, a flower mite that is monophagous on *H. patens* [35].

Within individuals, extended duration of flowering may be advantageous for spreading the risk of uncertain pollination, or reflect sparse or unpredictable resources in the understory [33]. In addition, reducing the number of flowers per day increases cross-pollination rates by promoting pollinator movement among plants [3]. Thus, individuals of plants with a continual or steady-state flowering strategy should be widely dispersed to promote the traplining behavior of their pollinators to increase outcrossing [53]. When individuals are clumped, however, pollinators can become territorial instead of traplining [53]. In the study sites I observed territorial behavior by hummingbirds on *H. patens*, and what appeared to be traplining behavior by euglossine bees, although I did not directly study euglossine bees and Malaise traps do not effectively capture euglossine bees. These observations suggest that *H. patens* or similar self-incompatible species [35] could have reduced fitness when planted in agricultural lands as a management action for biodiversity conservation. Although I did not compare fruit set rates among individuals in different land use types, fruit counts in *H. patens* supplemental patches averaged 198±20.1 ripe fruits per day (counted on one day per each month of 2008)- and many bird species were observed foraging on the fruits of *H. patens* in supplemental patches [29]. These observed fruit set rates were comparable to those reported from a more natural area in lowland Costa Rica- an average of 11–15 ripe fruits per infructescence produced over a two-wk period [54]. In this study I was not concerned though with the reproductive fitness of *H. patens*; instead, I wanted to understand the flower-visiting community response to resource predictability and whether this could be an effective management action for biodiversity conservation in agricultural lands.

A somewhat controversial term, keystone species can potentially be identified as those species whose removal is expected to result in the loss of at least half the assemblage considered [55], while another method includes identifying those plant species with low consumer specificity [12]. In this study, if the flower-visiting assemblage represents the entire consumer assemblage, then the potential keystone role of *H. patens* can be evaluated. I did not conduct a removal experiment, but species richness of the entire flower-visiting community was only 21% higher in the experimental plots, far less than half of the assemblage. However, casual observation revealed that specificity of flower-visitors was extremely low: just in the study sites I observed at least 80 species of arthropods and 9 hummingbird species foraging on floral resources and 15 bird species foraging on fruit resources. The identification of keystone or strong interactor species has long held appeal for restoration and conservation applications [55]. Menz and colleagues [17] suggest that the highest priority of plant species for restoration includes species that support a large number of pollinator species. This is especially true where the amount of space available for conservation actions is constrained, such as in small reserves or agroecosystems. If strong interactor plant species were preferentially planted in agroecosystems to enhance their conservation value, then perhaps fewer plants and plant species would be needed to obtain a conservation benefit. This management action would be more likely to be implemented by farmers reluctant to dedicate land to non-crop species. Identification of strong interactor species has proven difficult, however, in the Neotropics a good starting point is with the few plant species that have a steady-state flowering/fruiting phenology. The following are examples of plant families that are likely to have some shrub species with continual or steady-state phenology in the Neotropics: for both flowers and fruit resources- Rubiaceae (In Bawa et al. [34] four of 15 of the continually flowering species belonged to the Rubiaceae), Onagraceae (*Fuchsia* spp.), Melastomataceae, Annonaceae; for flower resources only- Verbenaceae (*Stachytarpheta* spp.), Lamiaceae. Future studies should aim to evaluate other species that produce steady-state food resources in different aseasonal environments to test whether the flowering/fruiting phenology has broad applicability for biodiversity conservation.

Finally, a recent meta-analysis aimed at understanding why agri-environmental measures vary in their effectiveness for pollinators found that measures were more effective when they were implemented in structurally simple versus cleared or complex landscapes, and when they increased contrast in floral resource availability compared to surrounding lands [56]. This study was conducted in the Monteverde region of Costa Rica, an important conservation area, and thus the landscape is structurally complex with high floral resource availability in the matrix surrounding the coffee agroforests. Therefore, the results of this study suggest that in more simplified landscapes and in landscapes where agriculture is more intensive, intercropping with plant species with a steady-state flowering phenology could be an even more effective management action for conservation of the flower-visiting community.

## Supporting Information

Figure S1 Map of the study area. White circles depict coffee agroforests that received the treatment of a supplemental patch of steady-state floral resources and black circles depict control farms.
(DOCX)

Table S1 List of all plant species occurring in the coffee agroforests, excluding herbaceous ground cover species.
(DOCX)

Table S2 AIC scores and likelihood ratio tests comparing models for significance of X,Y coordinates.
(DOCX)

Table S3 Model results for treatment effect, after adding X,Y coordinates, and site included as a random variable.
(DOCX)
