# Supplementary figures and images for: Intercropping with Shrub Species That Display a ‘Steady-State’ Flowering Phenology as a Strategy for Biodiversity Conservation in Tropical Agroecosystems
Source: PLoS One. 2014 Mar 5;9(3):e90510. doi: 10.1371/journal.pone.0090510 (PMC3943958; doi:10.1371/journal.pone.0090510)

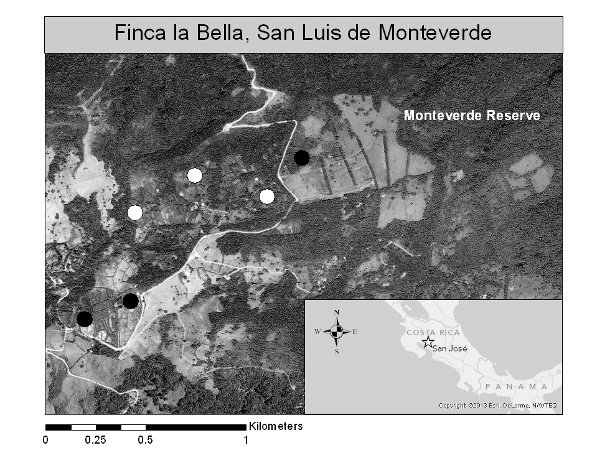

Supplement: Figure S1 — Map of the study area. White circles depict coffee agroforests that received the treatment of a supplemental patch of steady-state floral resources and black circles depict control farms. (DOCX) [file pone.0090510.s001.docx]
