# Supplementary material for: Intercropping with Shrub Species That Display a ‘Steady-State’ Flowering Phenology as a Strategy for Biodiversity Conservation in Tropical Agroecosystems
Source: PLoS One. 2014 Mar 5;9(3):e90510. doi: 10.1371/journal.pone.0090510 (PMC3943958; doi:10.1371/journal.pone.0090510)
Supplement: Table S1 — List of all plant species occurring in the coffee agroforests, excluding herbaceous ground cover species. (DOCX) [file pone.0090510.s002.docx]

**Table S1. List of all plant species occuring in the coffee agroforests, excluding herbaceous ground cover species.**

| Plant Species | H+ | H+ | C | H+ | C | C |
| --- | --- | --- | --- | --- | --- | --- |
| *Acnistus arborensens* | × | × | × | × | × |  |
| *Annona pruinosa* |  |  |  |  |  | × |
| *Bilia columbiana* |  | × | × | × |  |  |
| *Bursera simarouba* |  |  |  |  | × |  |
| *Casimiroa edulis* |  |  |  |  |  | × |
| *Cecropia obtusifolia* | × |  |  | × | × | × |
| *Cestrum racemosum* |  |  |  | × |  |  |
| *Citharexlym donnell-smithii* |  |  |  | × |  |  |
| *Chrysophyllum brenesii* |  | × | × | × |  |  |
| *Citrus spp.* | × | × | × | × | × | × |
| *Cordia eriostigma* | × |  |  | × |  | × |
| *Croton draco* | × | × |  |  |  |  |
| *Croton niveus* | × | × | × |  | × | × |
| *Cupania glabra* | × |  |  |  |  | × |
| *Daphnopsis americana* | × |  | × |  | × |  |
| *Diphysa americana* |  |  | × |  | × |  |
| *Diospyrus digyna* |  |  | × |  |  |  |
| *Ehretia latifolia* |  |  | × | × | × | × |
| *Unidentified epiphyte spp.* | × |  |  | × |  |  |
| *Erybothria japonica* |  |  | × |  |  |  |
| *Erythrina lanceolata* |  |  | × |  | × | × |
| *Erythrina sp.* |  |  | × |  |  |  |
| *Eugenia guatamalensis* |  |  |  | × |  |  |
| *Eugenia jambos* |  |  |  |  |  | × |
| *Ficus costaricana* | × |  | × |  |  |  |
| *Ficus lastericye* |  | × |  | × |  |  |
| *Ficus pertusa* |  | × |  | × |  | × |
| *Guazuma ulmifolia* | × |  |  |  |  |  |
| *Hamelia patens* | × | × |  | × | × |  |
| *Inga mortoniana* |  |  |  | × |  |  |
| *Inga punctata* | × | × | × | × | × | × |
| *Inga tondunzii* |  |  |  | × |  | × |
| *Lasiantha fructosa* | × |  |  | × | × |  |
| *Lonchocarpus olingathus* | × |  | × |  |  |  |
| *Mangifera indica* |  |  | × |  |  |  |
| *Mistletoe spp.* | × | × | × | × | × | × |
| *Montanoa guatamalensis* | × |  | × |  | × | × |
| *Mortoniodendron costaricense* |  | × |  | × |  |  |
| *Musa spp.* | × | × | × | × | × | × |
| *Myrsina coriacea* |  |  |  |  |  | × |
| *Nectandra membranaceae* |  |  |  | × |  |  |
| *Nectandra salicina* |  | × |  |  |  |  |
| *Ocotea floribunda* |  |  |  | × |  |  |
| *Ocotea monteverdensis* |  | × |  | × |  |  |
| *Persea americana* |  | × | × | × | × | × |
| *Psidium guayaba* | × | × |  |  | × | × |
| *Sapium glandulosum* |  | × | × | × | × | × |
| *Spondias spp.* |  | × |  |  |  | × |
| *Souroubea loczyi* |  | × |  | × |  | × |
| *Trema micrantha* |  |  |  |  | × |  |
| *Trichilia martiana* |  |  |  |  |  |  |
| *Trichilia havenensis* |  |  | × |  |  | × |
| *Zanthoxylum fagara* | × | × | × |  |  |  |
| *Zanthoxylum monophyllum* |  |  | × |  |  |  |
| Total  (Excluding epiphytes) | 18 | 19 | 23 | 24 | 18 | 21 |
