# Supplementary material for: Intercropping with Shrub Species That Display a ‘Steady-State’ Flowering Phenology as a Strategy for Biodiversity Conservation in Tropical Agroecosystems
Source: PLoS One. 2014 Mar 5;9(3):e90510. doi: 10.1371/journal.pone.0090510 (PMC3943958; doi:10.1371/journal.pone.0090510)
Supplement: Table S2 — AIC scores and likelihood ratio tests comparing models for significance of X,Y coordinates. (DOCX) [file pone.0090510.s003.docx]

**Table S2. AIC scores and likelihood ratio tests comparing models for significance of x, y coordinates.**

| Response variable | Model | AIC | Likelihood ratio | *P* |
| --- | --- | --- | --- | --- |
| Butterfly species | Treatment | 33.93 |  |  |
|  | Treatment + Lat, Lon | 37.36 | 0.57 | 0.75 |
| Butterfly abundance | Treatment | 46.82 |  |  |
|  | Treatment + Lat, Lon | 46.00 | 4.83 | 0.09 |
| Wasp species | Treatment | 235.52 |  |  |
|  | Treatment + Lat, Lon | 236.93 | 2.59 | 0.27 |
| Wasp abundance | Treatment | 383.00 |  |  |
|  | Treatment + Lat, Lon | 382.42 | 4.63 | 0.10 |
| Hummingbird species | Treatment | 152.13 |  |  |
|  | Treatment + Lat, Lon | 155.11 | 1.02 | 0.60 |
| Hummingbird abundance | Treatment | 216.58 |  |  |
|  | Treatment + Lat, Lon | 219.72 | 0.86 | 0.65 |
