# Supplementary material for: Intercropping with Shrub Species That Display a ‘Steady-State’ Flowering Phenology as a Strategy for Biodiversity Conservation in Tropical Agroecosystems
Source: PLoS One. 2014 Mar 5;9(3):e90510. doi: 10.1371/journal.pone.0090510 (PMC3943958; doi:10.1371/journal.pone.0090510)
Supplement: Table S3 — Model results for treatment effect, after adding X,Y coordinates, and site included as a random variable. (DOCX) [file pone.0090510.s004.docx]

**Table S3. Model results for treatment, after adding X-Y coordinates, and site included as a random variable.**

|  | Mean ± SE | | Model | |
| --- | --- | --- | --- | --- |
| Response variable | H+*^a^* | C*^b^* |  | *P* |
| **Hummingbirds** |  |  |  |  |
| Species richness | 1.02 ± 0.13 | 0.32 ± 0.08 | Likelihood ratio | 0.009 |
| Abundance | 1.39 ± 0.26 | 0.35 ± 0.09 | Likelihood ratio | 0.013 |
| **Butterflies** |  |  |  |  |
| Overall species richness | 10.67 ± 0.93 | 8.33 ± 1.02 | GLM | 0.06 |
| Nectarivore species richness | 7.75 ± 0.83 | 5.42 ± 0.87 | GLM | 0.03 |
| Frugivore species richness | 2.92 ± 0.34 | 2.92 ± 0.34 | GLM | 1.00 |
| Overall abundance | 34.1 ± 4.4 | 44.2 ± 8.5 | LM | 0.40 |
| Nectarivore abundance | 15.8 ± 2.4 | 16.8 ± 5.1 | LM | 0.52 |
| Frugivore abundance | 18.2 ± 3.5 | 27.3 ± 4.6 | LM | 0.07 |
| **Wasps** |  |  |  |  |
| Morphospecies richness | 30.9 ± 3.6 | 27.3 ± 2.8 | LM | 0.06 |
| Abundance | 202 ± 46.0 | 122.1 ± 26.4 | LM | 0.047 |

*^a^*Coffee agroforests with supplemental *H. patens* patch

*^b^*Coffee agroforests without *H. patens*
